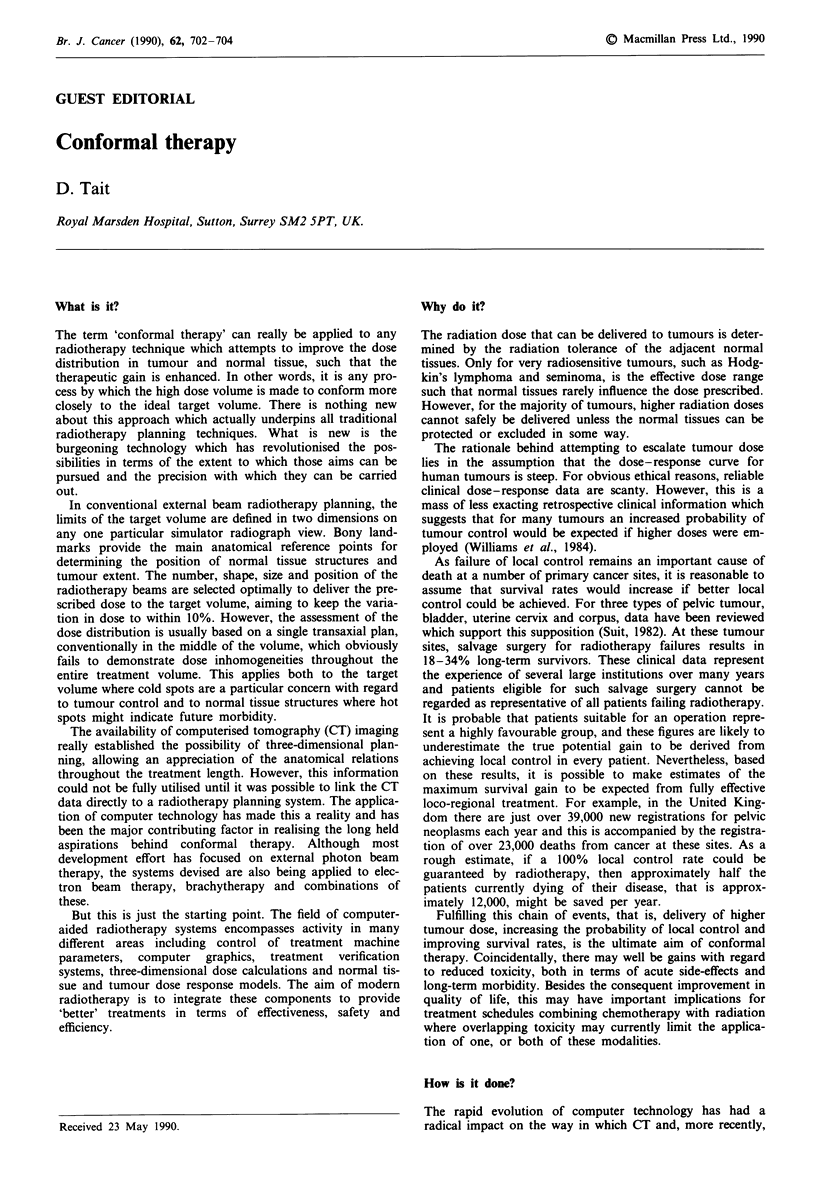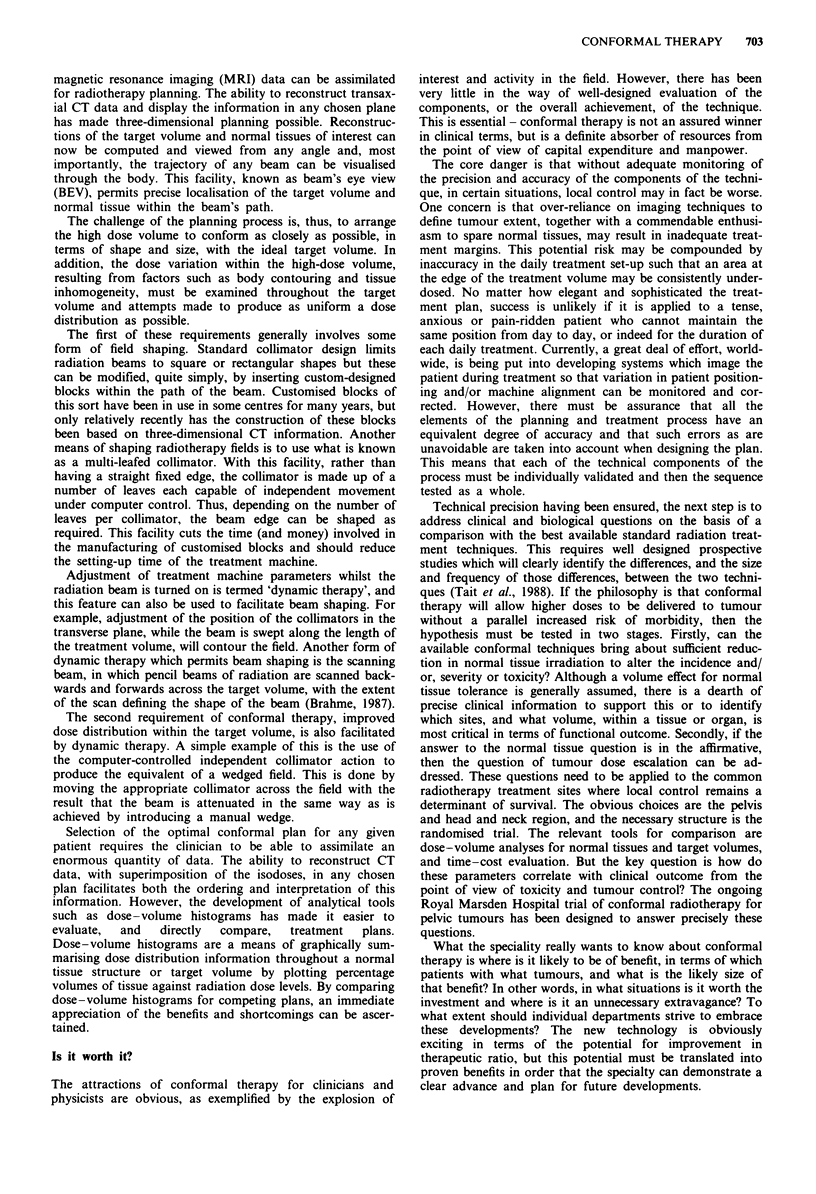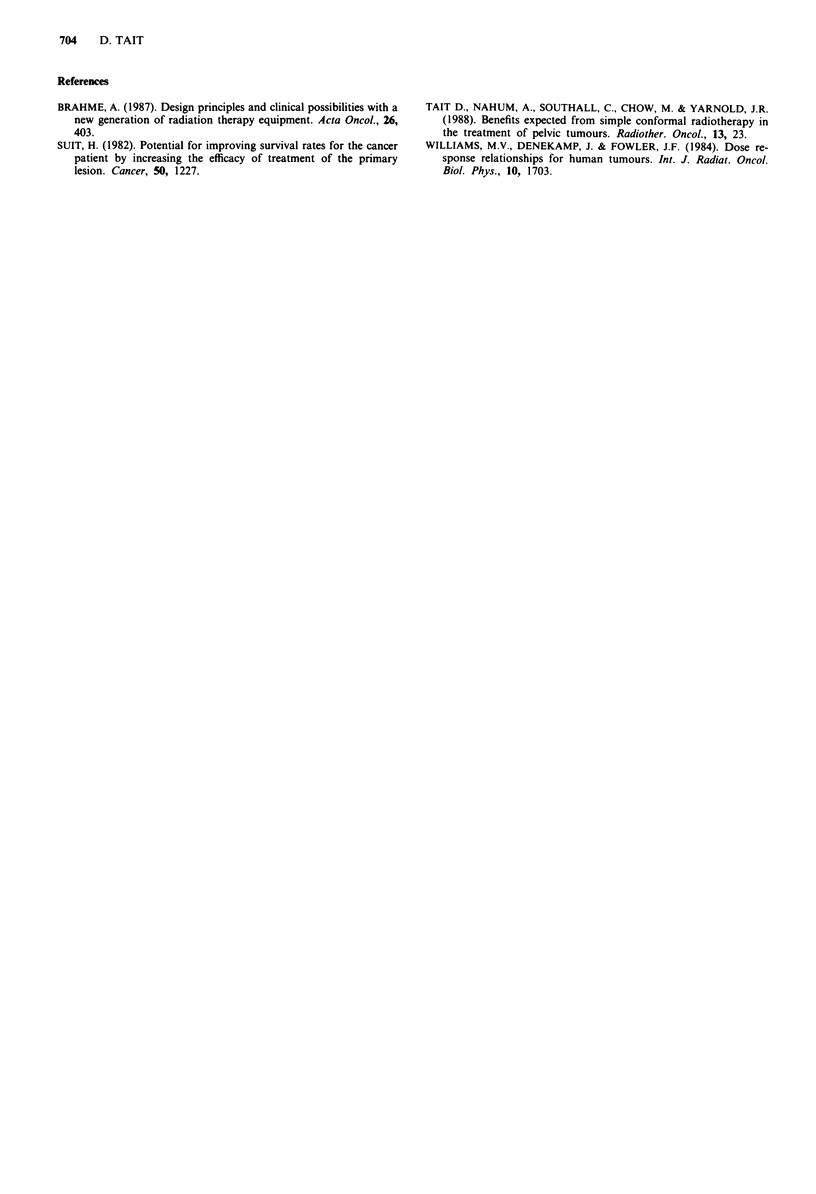# Conformal therapy.

**DOI:** 10.1038/bjc.1990.362

**Published:** 1990-11

**Authors:** D. Tait


Br. J. Cancer (1990), 62, 702-704
© Macmillan Press Ltd., 1990

GUEST EDITORIAL

### Conformal therapy

D. Tait

*Royal Marsden Hospital, Sutton, Surrey SM2 5PT, UK.*

### What is it?

The term 'conformal therapy' can really be applied to any radiotherapy technique which attempts to improve the dose distribution in tumour and normal tissue, such that the therapeutic gain is enhanced. In other words, it is any process by which the high dose volume is made to conform more closely to the ideal target volume. There is nothing new about this approach which actually underpins all traditional radiotherapy planning techniques. What is new is the burgeoning technology which has revolutionised the possibilities in terms of the extent to which those aims can be pursued and the precision with which they can be carried out.

In conventional external beam radiotherapy planning, the limits of the target volume are defined in two dimensions on any one particular simulator radiograph view. Bony landmarks provide the main anatomical reference points for determining the position of normal tissue structures and tumour extent. The number, shape, size and position of the radiotherapy beams are selected optimally to deliver the prescribed dose to the target volume, aiming to keep the variation in dose to within 10%. However, the assessment of the dose distribution is usually based on a single transaxial plan, conventionally in the middle of the volume, which obviously fails to demonstrate dose inhomogeneities throughout the entire treatment volume. This applies both to the target volume where cold spots are a particular concern with regard to tumour control and to normal tissue structures where hot spots might indicate future morbidity.

The availability of computerised tomography (CT) imaging really established the possibility of three-dimensional planning, allowing an appreciation of the anatomical relations throughout the treatment length. However, this information could not be fully utilised until it was possible to link the CT data directly to a radiotherapy planning system. The application of computer technology has made this a reality and has been the major contributing factor in realising the long held aspirations behind conformal therapy. Although most development effort has focused on external photon beam therapy, the systems devised are also being applied to electron beam therapy, brachytherapy and combinations of these.

But this is just the starting point. The field of computer-aided radiotherapy systems encompasses activity in many different areas including control of treatment machine parameters, computer graphics, treatment verification systems, three-dimensional dose calculations and normal tissue and tumour dose response models. The aim of modern radiotherapy is to integrate these components to provide 'better' treatments in terms of effectiveness, safety and efficiency.

Received 23 May 1990.

### Why do it?

The radiation dose that can be delivered to tumours is determined by the radiation tolerance of the adjacent normal tissues. Only for very radiosensitive tumours, such as Hodgkin's lymphoma and seminoma, is the effective dose range such that normal tissues rarely influence the dose prescribed. However, for the majority of tumours, higher radiation doses cannot safely be delivered unless the normal tissues can be protected or excluded in some way.

The rationale behind attempting to escalate tumour dose lies in the assumption that the dose-response curve for human tumours is steep. For obvious ethical reasons, reliable clinical dose-response data are scanty. However, this is a mass of less exacting retrospective clinical information which suggests that for many tumours an increased probability of tumour control would be expected if higher doses were employed (Williams *et al.*, 1984).

As failure of local control remains an important cause of death at a number of primary cancer sites, it is reasonable to assume that survival rates would increase if better local control could be achieved. For three types of pelvic tumour, bladder, uterine cervix and corpus, data have been reviewed which support this supposition (Suit, 1982). At these tumour sites, salvage surgery for radiotherapy failures results in 18-34% long-term survivors. These clinical data represent the experience of several large institutions over many years and patients eligible for such salvage surgery cannot be regarded as representative of all patients failing radiotherapy. It is probable that patients suitable for an operation represent a highly favourable group, and these figures are likely to underestimate the true potential gain to be derived from achieving local control in every patient. Nevertheless, based on these results, it is possible to make estimates of the maximum survival gain to be expected from fully effective loco-regional treatment. For example, in the United Kingdom there are just over 39,000 new registrations for pelvic neoplasms each year and this is accompanied by the registration of over 23,000 deaths from cancer at these sites. As a rough estimate, if a 100% local control rate could be guaranteed by radiotherapy, then approximately half the patients currently dying of their disease, that is approximately 12,000, might be saved per year.

Fulfilling this chain of events, that is, delivery of higher tumour dose, increasing the probability of local control and improving survival rates, is the ultimate aim of conformal therapy. Coincidentally, there may well be gains with regard to reduced toxicity, both in terms of acute side-effects and long-term morbidity. Besides the consequent improvement in quality of life, this may have important implications for treatment schedules combining chemotherapy with radiation where overlapping toxicity may currently limit the application of one, or both of these modalities.

### How is it done?

The rapid evolution of computer technology has had a radical impact on the way in which CT and, more recently, magnetic resonance imaging (MRI) data can be assimilated for radiotherapy planning. The ability to reconstruct transaxial CT data and display the information in any chosen plane has made three-dimensional planning possible. Reconstructions of the target volume and normal tissues of interest can now be computed and viewed from any angle and, most importantly, the trajectory of any beam can be visualised through the body. This facility, known as beam's eye view (BEV), permits precise localisation of the target volume and normal tissue within the beam's path.

The challenge of the planning process is, thus, to arrange the high dose volume to conform as closely as possible, in terms of shape and size, with the ideal target volume. In addition, the dose variation within the high-dose volume, resulting from factors such as body contouring and tissue inhomogeneity, must be examined throughout the target volume and attempts made to produce as uniform a dose distribution as possible.

The first of these requirements generally involves some form of field shaping. Standard collimator design limits radiation beams to square or rectangular shapes but these can be modified, quite simply, by inserting custom-designed blocks within the path of the beam. Customised blocks of this sort have been in use in some centres for many years, but only relatively recently has the construction of these blocks been based on three-dimensional CT information. Another means of shaping radiotherapy fields is to use what is known as a multi-leafed collimator. With this facility, rather than having a straight fixed edge, the collimator is made up of a number of leaves each capable of independent movement under computer control. Thus, depending on the number of leaves per collimator, the beam edge can be shaped as required. This facility cuts the time (and money) involved in the manufacturing of customised blocks and should reduce the setting-up time of the treatment machine.

Adjustment of treatment machine parameters whilst the radiation beam is turned on is termed 'dynamic therapy', and this feature can also be used to facilitate beam shaping. For example, adjustment of the position of the collimators in the transverse plane, while the beam is swept along the length of the treatment volume, will contour the field. Another form of dynamic therapy which permits beam shaping is the scanning beam, in which pencil beams of radiation are scanned backwards and forwards across the target volume, with the extent of the scan defining the shape of the beam (Brahme, 1987).

The second requirement of conformal therapy, improved dose distribution within the target volume, is also facilitated by dynamic therapy. A simple example of this is the use of the computer-controlled independent collimator action to produce the equivalent of a wedged field. This is done by moving the appropriate collimator across the field with the result that the beam is attenuated in the same way as is achieved by introducing a manual wedge.

Selection of the optimal conformal plan for any given patient requires the clinician to be able to assimilate an enormous quantity of data. The ability to reconstruct CT data, with superimposition of the isodoses, in any chosen plan facilitates both the ordering and interpretation of this information. However, the development of analytical tools such as dose-volume histograms has made it easier to evaluate, and directly compare, treatment plans. Dose-volume histograms are a means of graphically summarising dose distribution information throughout a normal tissue structure or target volume by plotting percentage volumes of tissue against radiation dose levels. By comparing dose-volume histograms for competing plans, an immediate appreciation of the benefits and shortcomings can be ascertained.

### Is it worth it?

The attractions of conformal therapy for clinicians and physicists are obvious, as exemplified by the explosion of interest and activity in the field. However, there has been very little in the way of well-designed evaluation of the components, or the overall achievement, of the technique. This is essential – conformal therapy is not an assured winner in clinical terms, but is a definite absorber of resources from the point of view of capital expenditure and manpower.

The core danger is that without adequate monitoring of the precision and accuracy of the components of the technique, in certain situations, local control may in fact be worse. One concern is that over-reliance on imaging techniques to define tumour extent, together with a commendable enthusiasm to spare normal tissues, may result in inadequate treatment margins. This potential risk may be compounded by inaccuracy in the daily treatment set-up such that an area at the edge of the treatment volume may be consistently underdosed. No matter how elegant and sophisticated the treatment plan, success is unlikely if it is applied to a tense, anxious or pain-ridden patient who cannot maintain the same position from day to day, or indeed for the duration of each daily treatment. Currently, a great deal of effort, worldwide, is being put into developing systems which image the patient during treatment so that variation in patient positioning and/or machine alignment can be monitored and corrected. However, there must be assurance that all the elements of the planning and treatment process have an equivalent degree of accuracy and that such errors as are unavoidable are taken into account when designing the plan. This means that each of the technical components of the process must be individually validated and then the sequence tested as a whole.

Technical precision having been ensured, the next step is to address clinical and biological questions on the basis of a comparison with the best available standard radiation treatment techniques. This requires well designed prospective studies which will clearly identify the differences, and the size and frequency of those differences, between the two techniques (Tait *et al.*, 1988). If the philosophy is that conformal therapy will allow higher doses to be delivered to tumour without a parallel increased risk of morbidity, then the hypothesis must be tested in two stages. Firstly, can the available conformal techniques bring about sufficient reduction in normal tissue irradiation to alter the incidence and/or, severity or toxicity? Although a volume effect for normal tissue tolerance is generally assumed, there is a dearth of precise clinical information to support this or to identify which sites, and what volume, within a tissue or organ, is most critical in terms of functional outcome. Secondly, if the answer to the normal tissue question is in the affirmative, then the question of tumour dose escalation can be addressed. These questions need to be applied to the common radiotherapy treatment sites where local control remains a determinant of survival. The obvious choices are the pelvis and head and neck region, and the necessary structure is the randomised trial. The relevant tools for comparison are dose-volume analyses for normal tissues and target volumes, and time-cost evaluation. But the key question is how do these parameters correlate with clinical outcome from the point of view of toxicity and tumour control? The ongoing Royal Marsden Hospital trial of conformal radiotherapy for pelvic tumours has been designed to answer precisely these questions.

What the speciality really wants to know about conformal therapy is where is it likely to be of benefit, in terms of which patients with what tumours, and what is the likely size of that benefit? In other words, in what situations is it worth the investment and where is it an unnecessary extravagance? To what extent should individual departments strive to embrace these developments? The new technology is obviously exciting in terms of the potential for improvement in therapeutic ratio, but this potential must be translated into proven benefits in order that the specialty can demonstrate a clear advance and plan for future developments.

### References

BRAHME, A. (1987). Design principles and clinical possibilities with a new generation of radiation therapy equipment. *Acta Oncol.*, **26**, 403.

SUIT, H. (1982). Potential for improving survival rates for the cancer patient by increasing the efficacy of treatment of the primary lesion. *Cancer*, **50**, 1227.

TAIT D., NAHUM, A., SOUTHALL, C., CHOW, M. & YARNOLD, J.R. (1988). Benefits expected from simple conformal radiotherapy in the treatment of pelvic tumours. *Radiother. Oncol.*, **13**, 23.

WILLIAMS, M.V., DENEKAMP, J. & FOWLER, J.F. (1984). Dose response relationships for human tumours. *Int. J. Radiat. Oncol. Biol. Phys.*, **10**, 1703.